# A Qualitative Study of the Context of Child and Adolescent Substance Use Initiation and Patterns of Use in the First Year for Early and Later Initiators

**DOI:** 10.1371/journal.pone.0170794

**Published:** 2017-01-25

**Authors:** Sharon Kingston, Maya Rose, Julian Cohen-Serrins, Emily Knight

**Affiliations:** Psychology Department, Dickinson College, Carlisle, Pennsylvania, United States of America; University of Edinburgh, UNITED KINGDOM

## Abstract

Individuals who initiate substance use before high school are at higher risk of negative outcomes. Eighty-six young adults between the ages of 18 and 28 participated in semi-structured qualitative interviews focused on the circumstances surrounding participants’ first use of substances and their pattern of use in the year following initiation in order to investigate similarities and differences between early versus later initiators. Initiation and use among early initiators were more likely to be encouraged by poor parental monitoring or active facilitation of use by parents. Early initiators were more likely to report risky patterns of use such as daily use and using alone. The data suggest that interventions targeting this population should focus on improving parental monitoring and decreasing positive parental attitudes toward adolescent substance use and efforts to increase identification and intervention by middle school staff to reach youth from high-risk families.

## Introduction

Individuals who begin using psychoactive substances at an early age, typically defined as prior to age 13 or 14 [1,2] are at greater risk of negative psychosocial, educational and mental health outcomes than individuals who initiate substance use at a later age. These early initiators typically begin their substance use with alcohol, tobacco and marijuana and experience more pronounced individual and family risk factors for substance abuse and dependence than later initiators [3–8]. Despite a relatively robust literature on the negative outcomes related to early substance use initiation and the risk factors associated with early initiation there is a paucity of research on whether the context of initiation and the early trajectory of use differs between early versus later initiators.

Early initiation of alcohol, tobacco and marijuana use has been associated with an increased risk of substance misuse and negative outcomes associated with intoxication. Early initiators are more likely to report polysubstance use, more frequent use of substances, more frequent episodes of intoxication and are more likely to develop substance use disorders than later initiators [2,9–16]. Early initiators are also more likely to participate in risky behaviors while under the influence such as unsafe driving, becoming involved in a physical fight, and risky sexual behavior [2,9–11,17–20].

In addition to experiencing negative outcomes directly resulting from substance use, early initiators are more likely to exhibit poor mental health and social functioning. Early initiators exhibit increased rates of depressive symptoms and disorders, and are more likely to report suicidal ideation and attempts than later initiators [21–28]. Early initiation is associated with increased risk of perpetrating or being the victim of dating violence, delinquent behavior, decreased academic performance and missing days of school or work [13,19,29,30].

As might be expected, individuals who initiate substance use at an early age often experience numerous individual, family and peer risk factors associated with poor developmental outcomes. Individual characteristics related to early initiation include externalizing symptoms such as hyperactivity, impulsiveness, inattention and early aggressive behavior [4,5] and temperamental characteristics such as novelty seeking, sociability, activity, and for girls, increased frequency of negative emotions [4,6,7].

Early initiators report less optimal family and social environments than later initiators. Early initiators are more likely to be raised by a single mother or by couples who experience less positive marital adjustment and more marital disruptions [3,5,6]. Notably, parents of early initiators are more likely to engage in increased substance use and their children are more likely to judge that their parents approve of substance use [3,5,6]. Early initiators report higher levels of conflict with parents and less parental control than later initiators during early adolescence. These early adolescents also report that their peers exert a stronger influence on their behavior than their parents [6]. In terms of support from adults outside of their families, early initiators are less likely to have supportive relationships with teachers than later initiators [6,8].

While numerous individual and family factors related to early initiation of substance use have been identified, very little research has been done to identify situational factors involved in children and adolescents’ first non-medical use of psychoactive substances. Understanding early adolescents’ own perceptions of their motivations to initiate substance use, the situations in which they initiate substance use and their initial reactions to their first use can help us to better understand the risk factors mentioned above, provide additional insights into the differences between early and later initiators and assist in developing strategies to delay substance use initiation among high-risk early adolescents.

The current study investigated the social and environmental contexts of substance use initiation in order to identify the motivations that lead to substance use initiation, the interpersonal and environmental contexts involved in initiation and the pattern of substance use in the first year following substance use initiation. Particular emphasis was placed on investigating factors that encouraged or inhibited substance use in the first year following initiation. The study was designed to reveal similarities and differences in motivations and initiation contexts between early initiators, defined as individuals who initiated substance use before high school (prior to age 13 or 14), and later initiators.

## Method

### Participants

Data for the current study were collected as part of a mixed methods study of substance use initiation consisting of a qualitative interview and quantitative survey of lifetime and current psychoactive substance use conducted by the first author. Purposive sampling was used to recruit young adults who had used psychoactive substances not prescribed by a medical provider. The participants for the study were recruited from three distinct populations: (1) students taking introductory psychology classes at a small undergraduate liberal arts college, as these classes fulfill a graduation requirement, these students represent a cross section of students enrolled in the college (n = 32), (2) young adults from the community surrounding the college who were not enrolled in a college or university (n = 28) and (3) young adults from the community surrounding the college specifically recruited because they attended a 12-Step Recovery program such as Alcoholics Anonymous or Narcotics Anonymous at least once in their lives (n = 26). These three populations were intentionally included to recruit participants who represented a range of substance use histories and educational backgrounds. The resulting participants reported substance use patterns that ranged from minimal use defined as infrequent use beginning after high school graduation to substance dependence. Age of initiation ranged from age 7 to age 21.

Participants were eligible to participate in the study if they were between the ages of 18 to 28 and had ever used tobacco, alcohol or another psychoactive substance not prescribed to them by a doctor. Participants recruited from the introductory level psychology courses received course credit for their participation. Participants from the surrounding community were recruited through flyers distributed at local businesses and Craig’s List advertisements and received $25.00 for participation. Eighty-six young adults, mean age 21.46, SD = 2.65 were interviewed. Fifty-five percent (n = 47) were men and 45% (n = 39) were women. The sample was predominately European American (86%, n = 73) but included individuals from urban, rural and suburban areas with diverse socioeconomic backgrounds.

### Procedure

All study procedures were approved by the Dickinson College Internal Review Board. All participants provided informed consent for the study prior to being interviewed. Participants were given a written copy of the consent form and the consent form was read to participants by their interviewer. The reading of the consent form and the participants’ verbal consent were recorded on audiotape. No written consent was collected in order to protect participants' confidentiality. The Dickinson College IRB approved this consent procedure. Records of each participant’s identifying information were destroyed after the participant completed the study.

Interviews were conducted by the first author or trained research assistants. The first author and principal investigator of the study is a European American woman with doctorate in in clinical psychology. She is a faculty member at a college in the Northeastern United States with a specific interest and expertise in substance abuse prevention. The research assistants were male and female European American undergraduate students at the college. The research assistants interviewed all of the participants from group 1: students in introductory psychology classes because it was believed that college students might be uncomfortable discussing their substance use histories with a professor. Both the first author and the research assistants interviewed participants from group 2: the young adults not enrolled in a college or university. The first author interviewed all of the participants from group 3: participants who had attended 12-Step recovery programs because group 3 participants completed a supplemental interview describing their responses to 12-Step programs that the research assistants were not trained to conduct [31]. Data from this supplemental interview was not included in the current analysis. The principal investigator and research assistants did not have any prior relationship with study participants before the interviews and did not have additional contact with participants upon completion of the interview.

Interviews were conducted in a private room at the college using a semi-structured interview guide. Participants were asked to describe their first and second use of psychoactive substances and their typical pattern of use of psychoactive substances in the year following their first use. The guide included questions about factors that encouraged or inhibited substance use during the first year following initiation and focused heavily on the interpersonal context surrounding participants’ substance use during their first year of use. The interview protocols were developed by the first author and refined several times as the first 10 participants were interviewed to improve the clarity of the interview questions. Copies of the interview guide can be found in Appendix A.

The interviews lasted between 20 and 40 minutes. Upon completion of the interview portion of the study, participants completed the survey of lifetime and current psychoactive substance use. Data from this survey was not included in the current analysis. Participants were then given a list of resources for more information about substance abuse and local substance abuse treatment providers. All study procedures were approved by the Dickinson College Internal Review Board.

### Analysis

Content analysis was performed using a thematic analysis framework. This qualitative analytic method was chosen because it allows researchers to highlight similarities and differences across groups of participants. The researchers worked from an essentialist/realist perspective that assumes that the language used by participants reflects their experiences, meanings and realities [32].

The interviews were transcribed and then coded by the first author and two research assistants using MAXQDA 10 a qualitative research program. Codes were developed inductively by reading the transcripts multiple times and noting patterns of reported motivations, reactions and behavior described by participants. The codes were revised multiple times during test coding of three randomly selected interviews and throughout the data coding process. Thirty percent of the interviews were double coded. The interrater reliability for the presence of specific codes in each interview was measured. The average interrater reliability for the double coded interviews was 92% and ranged from 87% to 95%. The first author and at least one other member of the coding team reviewed discrepancies in the double coded interviews and revised discrepancies through consensus. Themes were identified by the first author and reviewed by the rest of the authors. Themes were identified at a semantic level in order to organize and summarize participants’ experiences and relate the themes to previous research on substance use initiation for the purpose of understanding their implications for substance abuse prevention efforts [32].

Themes identified in the interviews of early initiators, defined as individuals who initiated substance use prior to attending high school were compared to themes identified in the interviews of participants who initiated substance use in high school or later. The authors chose this definition for early use because participants more readily remembered their first use of psychoactive substances in the context of their grade in school rather than their age.

## Results

Forty-two percent (n = 36) were coded as early initiators, 31% (n = 11) of these initiated substance use as elementary school students (11 years old or younger) and 69% (n = 25) initiated as middle school students (age 12 to 14). Fifty-eight percent (n = 50) initiated in high school or later with 14% (n = 7) of later initiators initiating after high school graduation (age 18 and older) and the remainder initiating as high school students (age 14 to 18).

### Common Patterns of Initiation and Use for Both Early and Later Initiators

Two themes related to substance use initiation and use during the year following initiation for both early and later initiators were detected. The first theme was that substance use was viewed as a normal, pleasurable activity for adolescents. The second theme was that minimal efforts were made by parents or other adults in the community to curtail adolescent substance use and in fact, some parents and adults facilitated adolescent substance use.

#### Substance use as a normal adolescent behavior

The common initiation situations described by both early and later initiators indicate that substance initiation is viewed as a normal adolescent behavior that is expected to produce pleasurable physical and psychoactive effects. Participants were most likely to initiate substance use with alcohol although some participants initiated with tobacco or marijuana or a combination of alcohol and tobacco or alcohol and marijuana. None of the participants reported initiating with a substance other than alcohol, tobacco or marijuana. When asked about the activities they engaged in during and following their use of substances, participants most often reported that they “hung out” and talked to peers. Substance use appears to have been the primary activity during initiation instances and participants described it as the central focus of their activities rather than as substances being used to enhance other activities. One participant described his initiation experience as, *“Just having fun and acting goofy*, *nothing sexual*, *nothing violent just acting goofy I guess*.*”* (ID204, male, initiated after high school)

Many participants reported that their decision to use substances occurred spontaneously when the substance was offered to them. Although some of participants did experience some degree of encouragement or coercion by peers, the decision to use substances was rarely the result of strong persuasion or coercion by peers. Rather, participants reported that they simply wanted to fit in with the individuals who were offering them substances.

“*Participant: I didn’t say no because I wanted to fit in like I said before. I wanted to fit in and I didn’t want to seem like a party pooper*.

*Interviewer: Okay, what did you think the other people would think of you if you said no*?

*Participant: He’s boring, he’s not the party type, he shouldn’t be allowed back to no more frat parties if he wasn’t going to get high, drunk, you know. Things like that.*”

(ID215, male, initiated in high school)

The perception that substance use was necessary to fit in with peers was bolstered by participants’ beliefs that substance use was a normal activity or a rite of passage for their age with one participant saying: *“It seemed like the logical next step in high school*.*”* (ID29, male, initiated in high school). Participants also reported curiosity as a reason to try substances with one participant stating that he wanted to see for himself if substance use was harmful: *“Just wanted to know what it felt like*, *see what the big deal is*, *you know*, *see if it’s actually bad for you or whatever*.*”* (ID202, male, initiated in high school).

The common belief that substance use is a normal adolescent activity was supported by the fact that participants typically expressed neutral or positive opinions about initiating substance use. It was rare for participants to express feelings of nervousness, guilt or even ambivalence related to use. When describing the psychoactive effects of substances during their first use, participants usually reported pleasurable emotional and perceptual effects.

“*I mean it made me happy, any kind of stressful thing that I had on my mind was completely gone, and I wasn’t even thinking about it, I was just laughing and telling stories, and not worrying about you know, oh well this happened today at school and I know when I go home, I’m gonna be getting yelled at or something like that.*”

(ID221, male, initiated in middle school)

“*I was making a lot of connections with stuff. And I like to write music and stuff like that and I was hearing stuff. And I hear that anyway, I was just hearing it differently and it was more intense.*”

(ID303, male, initiated in middle school)

When participants did report negative effects of substance use these effects usually focused on unpleasant physical effects such as nausea or dizziness.

Participants were most likely to report using substances for a second time within three months after initiation with some using within one to two weeks. The most common reason for using substances in the first year following initiation was that participants enjoyed the effects of the substance, felt that substance use was a normal activity for people their age and to fit in with peers. The common perception among participants was that substance use was a normative expected behavior that also provided pleasant emotional and perceptual experiences.

#### Minimal efforts by parents and other community adults to curtail use

When parents became aware of participants’ substance use their responses varied from active facilitation of use, to expressing disapproval or punishing participants for use. Most commonly however, parents and guardians remained unaware of participants’ use in the first year. The most common strategies participants used to avoid getting caught were to avoid or minimize contact with parents and guardians while intoxicated. This was sometimes accomplished by sleeping over at a friend’s house. Participants who used this strategy reported that the parents of one or more of their friends allowed them to use substances or did not monitor their activities closely enough to detect use, implying that a lack of monitoring or approval of youth substance use by adults within communities can facilitate use independently of the effects of parental monitoring and parental attitudes towards substance use.

“*A lot of times I would sleep over at Bill’s place, cuz that was kinda like the place, like his parents didn’t care so we’d always have a bunch of people over there, a lot of times I just wouldn’t go home at night.*”

(ID218, male, initiated in middle school)

While many participants reported making some effort to conceal their substance use, some participants reported that they did not need to use a strategy to conceal their use. A number of participants reported that their parents and guardians approved of their use. One participant reported that his mother allowed him to smoke marijuana.

“*Participant: My mom just called me actually, she asked me was I smoking weed. I said yeah, but she said she already knew because my eyes were always red. I said I don’t smoke all the time though. She said I know you don’t smoke all the time but your eyes is red now, do you smoke? I told her yes she said why didn’t you tell me? I was scared because I didn’t want to get in trouble. She said well you can be open to me like that, because I just moved with her and we had a lot of privacy with each other*.

*Interviewer: So other than talking to you and telling you to be honest with her did she do anything else or have any other reaction*?

*Participant: Nah, she just, the only thing she really said was don’t do it in the house.*”

(ID215, male, initiated in high school)

Other participants reported that although their parents or guardians disapproved of substance use they were unable to recognize signs that their children were intoxicated or were not home often enough to detect even regular use. One participant who was living with a guardian reported; *“Well she’s my great-great aunt*, *so she’s an older woman*, *so she didn’t really know what was going on*.*”* (ID322, male, initiated in middle school).

### Differences Between Early and Later Initiators

While the common initiation contexts and pattern of use in the first year following initiation of early and later initiators displayed some similarities, three differences between the groups were detected: (1) Differences in social and environmental context; (2) Parental substance use facilitated substance use by early initiators; and (3) Early initiators were more likely to describe patterns of use associated with increased risk of poor outcomes such as daily use.

#### Social and environmental contexts of early versus later initiators

The situations commonly reported by early initiators could be described as more childlike than those described by later initiators. Early initiators were most likely to initiate substance use in the afternoon or evening after school rather than at night, the most common time for later initiators to use substances for the first time. Some early initiators reported playing with their friends after use. One participant who initiated in elementary school described what he and his friends did after he smoked marijuana for the first time: *“I think we went out and rode bikes and played*, *did kids’ stuff*.*”* (ID114, male, initiated use in elementary school).

Early initiators often reported initiating substances in small single sex groups or with one friend of the same sex. Later initiators more commonly reported that their first use occurred at large parties with peers of both sexes than early initiators. Early initiators more often initiated substance use in their own home or backyard than later initiators who were most often initiated substance use at a friend’s home or backyard. This pattern continued throughout the first year of use with early initiators using most commonly in their own home and later initiators using most commonly at a friend’s home. It appears that both the activities and the social and physical settings of substance initiation differ for early initiators compared to later initiators with early initiators incorporating substance use into a typical elementary or middle school social and environmental settings.

#### Parental use as a facilitator of early initiator use

Perhaps the most striking differences between early and later initiators are the differences in family influence on substance use initiation and use in the first year. While later initiators most commonly initiated use and continued use with same age or older peers, early initiators reported much more variety in their substance use partners. Early users also often initiated and used with same age and older peers but also reported initiation and use with older siblings and parents or guardians more often than later users. One early initiator describes using substances with his mother: *“She had a party and I was hanging out with her and drinking*, *smoking pot…with her*.*”* (ID322, male, initiated in middle school)

Early initiators like later initiators often obtained substances from friends but more frequently reported that they stole substances from parents or guardians than later initiators. In some cases, participants were able to steal substances on a regular basis because their parent was frequently intoxicated and unable to monitor their own supply of alcohol, tobacco or other drugs. One participant initiated substance use with his mother’s supply and escalated immediately to daily use by continuing to steal his mother’s alcohol and prescription drugs.

“*So I would just take it, put it in a water bottle, you know, take a whole fifth if I wanted to, she wouldn’t notice. So I would often go get drunk, not only that she was on a bunch of medicines, and I used to pop her oxycontins from her surgeries. And she was on some weight loss pills too, and I found a way to mix the weight loss pills by crushing them up, crushing up xanax and half of a klonopin, throwing it in orange juice.*”

(ID302, male, initiated in middle school)

Other participants reported that substances were supplied directly by parents or guardians. Early initiators more frequently reported that parents or guardians facilitated their substance use and more often named parents or guardians as influences that encouraged them to use substances in their first year of use than later initiators. One participant referred to her parents’ desire to maintain a relationship with her by supplying her with cigarettes: *“It was*, *oh yea I’ll buy ya cigarettes*, *you know the parent-friend*, *uh*, *they constantly like either had liquor or beer in there*, *um*, *they said they’re recovering but they’re not*, *they just self-medicate*.*”* (ID305, female, initiated in middle school)

#### Problematic substance use patterns

Early initiators more frequently reported problematic patterns of use including using substances alone, escalating to daily use and appearing to have less interest in activities such as getting good grades or participating in organized extracurricular activities that might inhibit substance use escalation than later initiators. Early initiators much more frequently reported that they initiated substance use alone rather than in social situations than later initiators. One early initiator described his pattern of marijuana use:

“*I started smoking alone but it was still, it, the social aspect of it was still there and it didn’t change but then I basically added on you know, smoking alone. Cause a lot of the times I would be kinda like depressed or feel bad or you know any kind of emotional thing that I had going on and ya know, when I’m not at my best I tend to not be around people. Just kinda wall myself off so I don’t, can’t think of a good term for it, so I don’t change people’s impressions of me. Cause I’m like frustrated or not in the best mood. I would basically go and smoke alone and that would mellow me out or get me back to that kind of baseline personality and I would go out and meet people and be social.*”

(ID228, male, initiated in middle school)

When describing their pattern of substance use in the first year of use early initiators more frequently reported that they escalated to daily use within the first year than later initiators.

“*I’d wake up, I’d get ready, you know, smoke cigarettes and then I’d smoke a bowl or whatever you know, get ready, end up going to school, and you know I’d be at school all day, sometimes I’d sneak out at lunch time, and I’d smoke a bowl and come back in and you know, and school would be over and I mean I functioned fine.*”

(ID305, female, initiated in middle school)

Inhibitors of use also varied across early and later initiators. Early initiators more frequently reported that the main inhibitor of use in the first year was lack of opportunity to use substances than later users. Later users often reported that other goals such as performing well academically or athletically and a fear of being caught were the main inhibitors of substance use in their first year of use. Therefore, later users may have been more likely to have formed attachments to prosocial groups and adopted conventional norms that served to lessen their use of substances.

## Discussion

The interviews with young adults revealed differences between early and later initiators in initiation contexts and the pattern of use in the first year. Early users, as expected, describe much more problematic attitudes toward substance use, more negative patterns of use in the first year and less attachment to prosocial goals [6]. The interviews with early initiators indicate that they are often initiating substance use in home environments where they are not being closely monitored by adults. This lack of monitoring allows them to use substances in their own home or backyard and to steal substances from their homes. In some cases, adults are actively facilitating use. These results help explain why substance use by parents is correlated with early initiation [5,6,33]. Further, the presence of substances in the home increases availability, parental intoxication interferes with the ability to recognize use in their children or monitor their activities and positive attitudes towards substance use leads some parents to actively facilitate their child’s use.

These results indicate that prevention and early intervention programs aimed at children who are at risk for early initiation or children and early adolescents who have already initiated substance use would benefit from a strong family component [34,35]. Parents who use substances need interventions that will allow them to develop skills to deliver strong anti-use messages to their children despite their own use, to secure and closely monitor the substances in their homes and to monitor the behavior of their children.

Although family-based prevention appears to be a promising strategy, the family contexts of some of the respondents indicate that not all families would be able to respond to such interventions. Parents who are dependent on substances other than tobacco likely face significant challenges in preventing use by their children. Parents who actively facilitate use by their children and adolescents clearly are unable or unwilling to prevent substance use in their children. Stronger efforts by middle school personnel to deliver effective prevention programs, to recognize the signs of intoxication and to support and intervene with children and adolescents who are at high risk for substance use or actively using substances appear to be needed to reach youth in these families [8].

The widely held view among both early and later initiators that substance use was a normal adolescent activity supports the theory that the social norms about adolescent substance use held by most participants and by some adults in their communities encourage substance use initiation during adolescence [36,37]. The extent to which both early and later initiators reported that their first use was unplanned and in response to the belief that substance use was normal adolescent behavior fits very closely with the Social Reaction Model of Health Risk [38,39]. This model, developed specifically to describe adolescents’ decisions to participate in risky behavior posits that many instances of adolescent risky behavior occur due to a willingness to participate in the behavior in social settings rather than a planned intention to seek out opportunities to engage in the behavior. Furthermore, the model posits that this willingness to engage in the behavior arises out of positive images of people who engage in such behaviors and feelings of being similar to people who engage in the behavior. The predominant pattern of initiation described by participants in the current study involved spontaneous decisions to use substances when they were offered in social settings and participants rarely described feelings of ambivalence, nervousness or guilt regarding using substances because they perceived that using substances was congruent with their image of typical adolescent behavior.

Prevention programs that include social norming interventions to decrease the perception that substance use is a normative behavior and teach adolescents how to refuse offers to use substances are strongly supported by the current study [40,41]. In addition, the study suggests that children and adolescents should be encouraged to engage in goal directed prosocial activities such as striving to succeed academically or in extracurricular activities in order to decrease motivation to use substances [42]. Later initiators often reported that they inhibited their first year use because they feared that use would interfere with important academic or extra-curricular goals.

Finally, the inability of many parents of both early and later users to detect substance use in the first year and the substantial proportion of parents who did not intervene firmly to curtail use when they did detect it, suggests the need for increased efforts to educate parents about the dangers of adolescent substance use and support parents in developing skills to set strong anti-use expectations, effectively monitor their children and enforce appropriate consequences for adolescent substance use. In addition, many participants reported that they were able to use substances as adolescents despite prohibitions by their own parents because other adults in the community tolerated or facilitated use. Prevention interventions aimed at decreasing support for adolescent substance use among adults and increasing effective monitoring among community adults may decrease opportunities for use in this population of adolescents [43].

The study had a number of limitations. One of the most prominent was the lack of racial and ethnic diversity in the sample. The study was conducted in an area of the United States with a predominately European American population and the participants recruited for the study reflected that demographic. Only 14% of participants identified as a race/ethnicity other than European American. Future studies should investigate the initiation contexts of adolescents from diverse racial and ethnic backgrounds. It is possible that there are racial and ethnic differences in the typical initiation scenarios among children and adolescents or in the reactions of parents who detect substance use by their children.

Another limitation to the study was the reliance on participants’ reports of their parents’ attitudes and behaviors. The participants’ descriptions of their parents’ reactions to their substance use paints a fairly strong picture of families and communities in which adolescent substance use is often expected, tolerated and in some cases facilitated by adults. It is important to remember however, that these descriptions are the perceptions of the participants and not direct reports from parents and other community adults. While participants’ perceptions of their parents’ attitudes quite likely had a strong impact on their own attitudes towards substance use and their subsequent behavior, their parents may have described their attitudes and behaviors very differently. Future studies should include parents to better understand their attitudes towards substance use by their children and their strategies for dealing with this issue.

Finally, all of the data were retrospective reports of participants’ past experiences. Although participants were being asked to describe fairly salient experiences it is likely that they may not have accurately remembered all of the details of their first use and subsequent pattern of use.

Despite these limitations, the study provides a unique look at the situations of substance use initiation, helps explain the strong relationships between family risk factors and substance use among adolescents, supports prevention efforts based on social norms and suggests that greater efforts to support and protect children and adolescents at risk for early use are needed in schools and communities. The most striking finding of the study is the extent to which substance use is accepted or even facilitated by parents of some adolescents, particularly, early initiators. Initiatives to curtail these behaviors by parents should be incorporated into prevention programs.

## Appendix A

### Substance Use Initiation Interview

1. ***Have you ever used alcohol*, *tobacco*, *marijuana or another drug*? *Do not count drugs prescribed for you by a doctor or medical provider*.**
  **If yes**, continue to item 2.
  **If no**, say “So just to be clear, you have never had an alcoholic beverage like a beer, smoked a cigarette or used any other drug like marijuana. Is that true?”
    If the respondent replies that this is true, skip the interview portion of the study and begin the computer survey.
    If the respondent replies that he or she has used a drug in the past continue to item 2.
2. ***What was the first drug you ever used*?** (alcohol, tobacco, marijuana, another drug like cocaine, ecstasy, or a prescription drug like Ritalin that wasn’t prescribed for you)
3. ***Did you use more than one drug at that time*? *By that I mean during the same evening or afternoon*, *party etc*.**
  **If yes**, What other drugs did you use?
  **If not** skip to item 4.
4. ***Please describe as much as you can remember about the circumstances of your first use of ______________ (substance named by participant)*. *Include as many details as you can remember*.**
  Prompts:
  How old were you? What grade were you in? Where were you? Who were you with? Were they boys or girls? Were they the same age as you? Older? Younger? What was the time of day? What was the time of year? How did you get the __________________? Were you planning on using ____________ or did it happen spontaneously? How much ________________ did you use/drink/smoke?
  **Ask any prompt questions not covered in participant’s account. Summarize what the participant has just told you and ask if he or she has anything to add.**
5. ***How did you feel about using _____________*? If the participant answered this question clearly in their description of the event, summarize what they said and ask if they felt any other emotions.**
  If the participant has trouble, prompt (nervous, excited, happy, scared, you did not feel very much—it was no big deal)
6. ***Why did you use the ________________*? If the participant answered this question clearly in their description of the event, summarize what they said and ask if they had any other reasons for using. If the participant gives more than one reason, repeat the reasons and ask if one reason was primary or stronger than the others.**
7. ***What effect did the _________________have on you*?**
  If the participant has trouble, prompt (did you get drunk or high, did you feel anything)
8. ***Did you enjoy the effect of the ____________________*?**
9. ***What did you do while using __________________*? *What did you do after using _____________________*?**
  If the participant has trouble, prompt (hang out with friends, dance, romantic activity)
10. ***After you first used _____________*, *when did you use _______________ or another drug again*? *How long did you wait before your second use*?**
11. ***What drug did you use the second time you used drugs*?**
12. ***Please describe as much as you can remember about the circumstances of your second use of drugs*. *Include as many details as you can remember*.**
  Prompts:
  How old were you? What grade were you in? Where were you? Who were you with? Were they boys or girls? Were they the same age as you? Older? Younger? What was the time of day? What was the time of year? How did you get the __________________? Were you planning on using ____________ or did it happen spontaneously? How much ________________ did you use/drink/smoke?
  **Ask any prompt questions not covered in participant’s account. Summarize what the participant has just told you and ask if he or she has anything to add.**
13. ***Think about your drug use history*. *Describe your use of alcohol*, *tobacco and other drugs in the* YEAR *following your first use*.**
  Prompts:
  On average, how often did you use drugs? Where did you use them? How did you usually get them? Who did you use them with? Why did you usually use them?
  **Ask any prompt questions not covered in participant’s account. Summarize what the participant has just told you and ask if he or she has anything to add.**
14. **If participant stopped using drugs in the year following their first use: *What factors led you to abstain from using drugs during that first year*?**
15. ***Were there instances when you wanted to use drugs but didn’t*? *What stopped you from using*?**
16. ***What encouraged you to use drugs during that first year*?**
17. ***What enabled you to use drugs on occasions when you did use drugs*? *What stopped you from getting caught*? *What strategies did you use to stop from getting caught*?**
  **If person got caught:**
  ***What happened*? *Did getting caught effect your drug use in any way*?**
18. ***Have you had any negative experiences while using drugs*?**
  **If yes:**
  ***What happened*? *Did this experience affect your drug use in anyway*? *How*?**
19. ***Is there anything else about your drug use that you would like to share or think would be important for us to know about*?**

***We are done with the interview portion of the study*. *Now I am going to ask you to answer some survey questions*. *The questions will ask for a more detailed drug use history and some questions about your risk perceptions regarding use of certain drugs*. *Remember everything you tell us is anonymous***.
